# Vigorous regular leisure-time physical activity is associated with a clinically important improvement in back pain – a secondary analysis of randomized controlled trials

**DOI:** 10.1186/s12891-021-04727-2

**Published:** 2021-10-08

**Authors:** Lena W. Holm, Clara Onell, Martin Carlseus, Robin Ekwurtzel, Olle Holmertz, Tony Bohman, Eva Skillgate

**Affiliations:** 1 Institute of Environmental Medicine, Unit of Intervention and Implementation Research for Worker Health, Karolinska Institutet, Stockholm, Sweden; 2 Department of Health Promotion Sciences, Musculoskeletal & Sports Injury Epidemiology Center, Sophiahemmet Högskola, Box 5605, 114 86 Stockholm, Sweden; 3 Faculty of Medicine, Uppsala University, Uppsala, Sweden; 4 School of Health and Welfare, Dalarna University, Falun, Sweden

**Keywords:** Physical activity, Musculoskeletal pain, Epidemiology/survey research

## Abstract

**Background:**

Neck and back pain are musculoskeletal conditions with serious individual and societal consequences. Current evidence about the prognostic value for neck and back pain is limited and conflicting. This prospective cohort study aimed to assess the association between leisure-time physical activity (LPA) and improvement of neck and/or back pain in a working population receiving manual therapy or general care in one of two randomized controlled trials (RCTs).

**Methods:**

Analyses of data from two RCTs evaluating the effect of manual therapies for neck and/or back pain was conducted. Participants (*n* = 1 464) answered questionnaires about frequency and effort level of LPA at baseline. LPA on moderate or vigorous levels was compared to no or low/irregular moderate and vigorous levels. Pain intensity was assessed with numerical scales at baseline and 3-, 6-, and 12-month follow-up. The outcome was minimal clinically important improvement in pain intensity, defined as ≥2 points improvement in mean pain intensity at follow-up. Crude- and adjusted risk ratios (RR) with 95% confidence intervals (CI) were calculated with Poisson regression analysis and stratified by pain location.

**Results:**

Participants with neck and/or back pain performing vigorous LPA showed a minimal clinically important improvement after 12 months compared to the control group; RR 1.35 (95% CI; 1.06-1.73). No effect was observed at 3 or 6 months. Moderate LPA did not improve pain intensity in any follow-up. Stratified analyses revealed that the effect of vigorous LPA at 12 months in back pain was RR 1.83 (95% CI; 1.26-2.66) and neck pain RR 1.06 (95% CI; 0.75-1.49).

**Conclusions:**

Persons with neck and/or back pain receiving manual therapy or general evidence-based care have greater chance of improvement after 12 months if they prior to treatment frequently practice vigorous LPA. When analyzed separately, the effect was only present for back pain.

**Trial registration:**

Registration in Current Controlled Trials (ISRCTN), Stockholm Manual Intervention Trial (MINT), ISRCTN92249294 BJORN-trial, ISRCTN56954776

## Background

Neck- and back pain (NBP) are the most common musculoskeletal conditions worldwide and dominant causes of years lived with disability in high- and middle-income countries [1]. The global lifetime prevalence of non-specific neck pain is estimated to 50% and non-specific back pain to 80%, and these conditions often occur concurrently [2–4]. Consequently, NBP is a major reason for sick leave and associated with individual and socioeconomic costs [5].

Several modifiable and non-modifiable factors may play a role for the prognosis of NBP, although summarized evidence is limited in systematic reviews [6, 7]. Physical activity may have multiple physiological effects on pain and could be of prognostic value in NBP recovery [8, 9]. Among others, exercise increases angiogenesis and blood flow which increases oxygen, nutrients and removal of waste products in affected tissues [8, 9]. Moreover, endurance exercise is associated with release of endogenous β-endorphins affecting pain and mood processing [10]. In a cross-sectional study by Hansen et al., leisure-time physical activity (LPA) among workers was associated with higher saliva cortisol levels and self-perceived energy [11].

Despite known biological mechanisms as potentially beneficial for NBP recovery, there is conflicting evidence from the etiological literature of the prognostic value of physical activity. However, in earlier studies, we found that LPA was of moderate prognostic value for recovery from long-duration NBP in women, but not in men [12, 13]. One of them [12] was also included in a systematic review on LPA as a prognostic factor for back pain (BP) where, however, the majority of included studies showed no effect. Only one study concluded that moderate and vigorous LPA decreased pain intensity and disability over 12 months in patients with persistent BP [14]. The authors concluded that there is low quality evidence that LPA may *not* be a prognostic factor for BP. In an overview of systematic reviews by Walton et al. on prognostic factors in NP, two systematic reviews reporting on LPA were included, concluding that there was moderate evidence of no effect of LPA on NP recovery [7].

Thus, although health benefits of physical activity are well-established, current evidence about the prognostic value for NBP is limited and conflicting. This study aimed to assess the association between moderate and vigorous regular LPA and the minimal clinically important improvement of pain intensity (MCII) in a working population with NBP, who have received manual therapy or general evidence-based care in one of two randomized controlled trials (RCTs). Furthermore, the aim was to assess if pain location is an effect modifier, i.e. if the association between LPA and MCII is different for participants with NP and BP, respectively. This was done due to the inconsistency and conflicting results in previous studies on NP and BP [7, 12–16].

## Methods

All methods in the study were carried out in accordance with the Helsinki guidelines and declaration or any other relevant guidelines.

### Study design

This prospective cohort study aimed to assess the association between the exposure leisure-time physical activity (LPA) and the outcome improvement of neck and/or back pain in a working population receiving manual therapy or general care in one of two RCTs. It is an observational study based on secondary analyses of two RCTs aiming to compare the effect of therapies for NBP. The Stockholm Manual Intervention Trial (MINT) started in 2010 and compared three combinations of manual therapy. The BJORN-trial started in 2005 and compared manual therapy with general evidence-based care including advice to stay active and pain coping strategies. Description of the trials are found in detail elsewhere [17, 18].

### Study population

Participants in the MINT (*n* = 1 057) were recruited when seeking care at the Scandinavian College of Naprapathic Manual Medicine. In the BJORN-trial, participants (*n* = 409) were recruited through two large public companies based in Stockholm.

Inclusion criteria for the present study were age 18-65 years, NP (neck/shoulder pain and/or upper back pain above the 11^th^ thoracic vertebrae, with or without pain in upper extremities and chest) and/or BP (including pain below the 10^th^ vertebrae and/or gluteal area with or without pain in lower extremities) causing marked dysfunction, as well as available information about LPA.

Exclusion criteria were not mastering the Swedish language, pregnancy, current or previous cancer, contraindication for spinal manipulation, spinal stenosis, ‘red flags’ (i.e. older than 55 when pain debuted, recent trauma in pain location, consumption of steroids, obvious structural deformity of the spine, saddle anesthesia/sphincter disturbance, inflammatory or rheumatic diseases, peripheral joints affected). Further description is found in the original articles [17, 18].

### Data collection

Self-administrated questionnaires were distributed at baseline and at follow-ups at 3, 6- and 12 months, respectively. For the present study, we considered the questions phrased in the same way in both the MINT and the BJORN-trial. Variables for descriptive and analytic purposes included age, sex, education, body mass index, daily smoking, previous episodes of pain, pain location, duration of current pain and pain-related disability [18, 19].

A modified version of the Chronic Pain Questionnaire (CPQ) was used to assess pain intensity [20]. The CPQ includes three questions about pain intensity (current pain intensity, average and worst pain intensity) measured on 11-point numerical rating scales. The questions were modified to include episodes of pain during the past four weeks instead of past six months.

### Exposures

Effort level and frequency of LPA were assessed through a valid questionnaire where LPA was defined as recreational, sporting or outdoor activities exceeding 20 minutes per occasion [21]. Participants were asked to report their frequency of low exertion LPA (walks and bike riding), medium exertion LPA (effort where you can keep a conversation with somebody) and high exertion LPA (high pulse, high effort) as a) never, b) irregularly, c) once per week, d) twice per week or e) ≥3 times per week. They were categorized as exposed or unexposed and analyzed accordingly: (i) participants reporting frequency level c-e) on a moderate level and/or d) on vigorous level or (ii) participants reporting frequency level e) on a vigorous level with or without LPA on other levels were defined as exposed to “moderate” and “vigorous” LPA, respectively. Exposed participants (i.e. two levels of exposure) were independently compared to unexposed participants, i.e. those engaging in only low effort level LPA and/or no/irregular moderate and vigorous LPA, hence categorized as the control group.

### Outcome

The outcome of interest was MCII assessed with the CPQ at follow-ups. Differences in pain intensity between baseline and follow-ups were calculated based on a mean score of the pain intensity questions from the CPQ [20]. Answers were dichotomized into having MCII or not based on whether the CPQ score was improved by ≥2 points or not [22–24].

### Confounders

Based on existing literature on the topic and on clinical experience and after considering potential mediators and colliders, we accounted for sex, age, body mass index, daily smoking, level of education, pain intensity at baseline, pain-related disability at baseline, pain duration at baseline, previous episodes of pain and pain location, as described in Table 1.

**Table 1:** Potential confounders considered in the analyses

Potential confounder	Description
Sex	Male, female
Age	Continuous
Body mass index	<18.5, 18.5-24.9, 25-30, >30 continuous
Daily smoking	Yes, no
Level of education	Elementary (1-9 y), secondary (10-12 y), university (13-16 y), higher academic education (>16 y)
Pain intensity at baseline	Mean score on the Chronic Pain Questionnaire based on current pain intensity, worst pain intensity during the last 4 weeks and average pain intensity during the last 4 weeks, continuous
Pain-related disability at baseline	Mean score on the Chronic Pain Questionnaire based on how pain hindered daily, recreational-, social-, and family activities as well as interfered with work during the last 4 weeks, continuous
Pain duration at baseline	<1 month, 1-3 months, 4-6 months, >6 months
Previous episodes of pain	Yes, no
Pain location	Neck pain, back pain or equally bad pain in both locations

### Statistical analysis

This paper is based on data from two RCTs but does not use the RCT design. Instead, it is an observational study. Descriptive data is presented stratified on levels of LPA. Also, a lasagna plot illustrating outcome patterns among participants responding to all follow-ups was computed [25]. Generalized linear models with Poisson regression was used to assess associations between moderate and vigorous levels of LPA and MCII at 3, 6 and 12 months. Risk ratios (RR) with 95% confidence intervals (CI) were calculated, where RR>1 denotes that the exposure is favorable for the outcome. First, one crude model for each follow-up was computed. Thereafter, one variable at a time was added to each model. If the beta estimate of the exposure variable changed by 5% or more when introducing the variable, it was considered a confounder and was included in the final adjusted model [26]. Since data collection of the MINT started five years later than the BJORN-trial and had different recruitment bases, we also included a variable indicating the five treatment arms (two in the BJORN -trial and three in MINT) in the final models. This was done to control for potential differences in the study populations, not captured by the variables in the confounding control. In addition, as a sensitivity analysis and to assess consistency of results over the whole follow-up period, participants reporting MCII at all follow-ups were compared to those who did not report MCII at any of the follow-ups. This analysis was also made with Poisson regression including confounding control and reported with RR with 95% CI.

We also stratified the analysis in two groups based on pain location to (i) mainly NP (*n* = 806) and (ii) mainly BP (*n* = 526) or equally disturbing pain in neck and back (*n* = 128). These analyses were also controlled for potential confounders as explained above.

Analyses were performed with SPSS for Windows version 26 (®IBM statistical software) except the lasagna plot which was performed in Microsoft Excel version 1910.

### Ethical permission

Ethical permission was received by the Ethical Review Board of Stockholm (Stockholm Manual Intervention Trial (MINT), d.nr. 2009/1848-31/2, BJORN-trial, d.nr. 03-657 and 2014/190-32). Participants gave their informed consent prior to data collection. Personal rights were protected.

## Results

### Study population

**Fig. 1:**
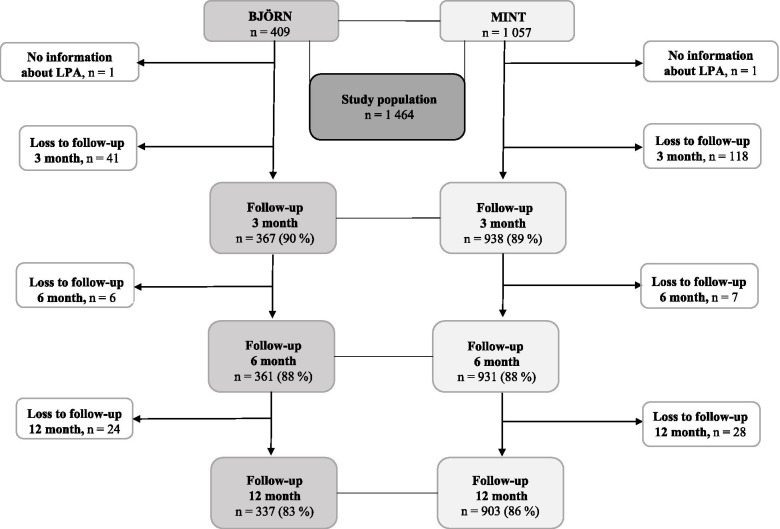
Flowchart of study population and follow-up. Abbreviations: LPA leisure-time physical activity

One thousand four hundred and sixty-six participants were eligible for inclusion, as illustrated in Fig. 1. Two participants were excluded prior to analysis due to missing information about their LPA. Thus, the study population consisted of 1 464 participants. The follow-up rate varied from 83-90%.

Table 2 summarizes baseline characteristics stratified by level of LPA. The mean age was 38.6 years and 71% were female. Most of the participants had an ongoing pain episode for either less than one month or more than six months at baseline, and 80% reported that they had experienced previous episodes of pain.

**Table 2:** Baseline characteristics categorized by level of leisure-time physical activity

Variables	No/irregular LPA^**a**^	Moderate LPA^**b**^	Vigorous LPA^**c**^	All
**All, n (%)**	350 (24)	808 (55)	306 (21)	1 464 (100)
**Sex, n (%)**				
Male	103 (29)	206 (25)	123 (40)	432 (30)
Female	247 (71)	602 (75)	183 (60)	1 032 (70)
**Age, mean years ± SD**	42.2 **±** 13.0	38.4 **±** 12.4	35.1 **±** 11.6	38.6 **±** 12.6
**Level of education, n (%)**				
Elementary (1-9 y)	32 (9)	38 (5)	17 (5)	87 (6)
Secondary (10-12 y)	144 (41)	285 (35)	103 (34)	532 (36)
University (13-16 y)	152 (43)	389 (48)	146 (48)	687 (47)
Higher academic education (>16 y)	22 (7)	96 (12)	40 (13)	158 (11)
**Body mass index, n (%)**				
<18.5	6 (2)	9 (1)	3 (1)	18 (1)
18.5-24.9	206 (59)	504 (63)	201 (66)	911 (63)
25-30	99 (28)	211 (26)	87 (28)	397 (27)
>30	38 (11)	80 (10)	15 (5)	133 (9)
**Daily smoking, n (%)**				
Yes	69 (20)	108 (13)	42 (14)	219 (15)
No	281 (80)	700 (87)	264 (86)	1 245 (85)
**Pain duration this episode, n (%)**				
<1 month	103 (30)	280 (35)	134 (44)	517 (36)
1-3 months	56 (15)	151 (19)	56 (18)	263 (18)
4-6 months	30 (9)	63 (7)	27 (9)	120 (8)
> 6 months	160 (46)	313 (39)	89 (29)	562 (38)
**Previous episodes of pain, n (%)**				
Yes	287 (82)	649 (80)	233 (76)	1 169 (80)
No	63 (18)	159 (20)	73 (24)	295 (20)
**Pain location, n (%)**				
Neck	203 (58)	456 (57)	147 (48)	806 (55)
Back	114 (33)	282 (35)	130 (43)	526 (36)
Neck and back	31 (9)	70 (8)	27 (9)	128 (9)
**Pain intensity, mean score ± SD**^**d**^	5.6 **±** 1.7	5.5 **±** 1.6	5.4 **±** 1.7	
**Pain related disability, mean score ± SD**^**d**^	2.7 **±** 2.3	2.7 **±** 2.2	2.4 **±** 2.2	

*Abbreviations*: *LPA* leisure-time physical activity

^a^No/irregular moderate or vigorous LPA, but any on low exertion level (walks and bike riding)

^b^Regular medium exertion level (effort where you can keep a conversation with somebody) up to three times/week or more, and/or once or twice/week on a high exertion level (high pulse, high effort)

^c^Regular vigorous LPA three times or more/week with or without LPA on low or moderate level

^d^Pain intensity and pain related disability at baseline assessed with Chronic Pain Questionnaire

**Fig. 2:**
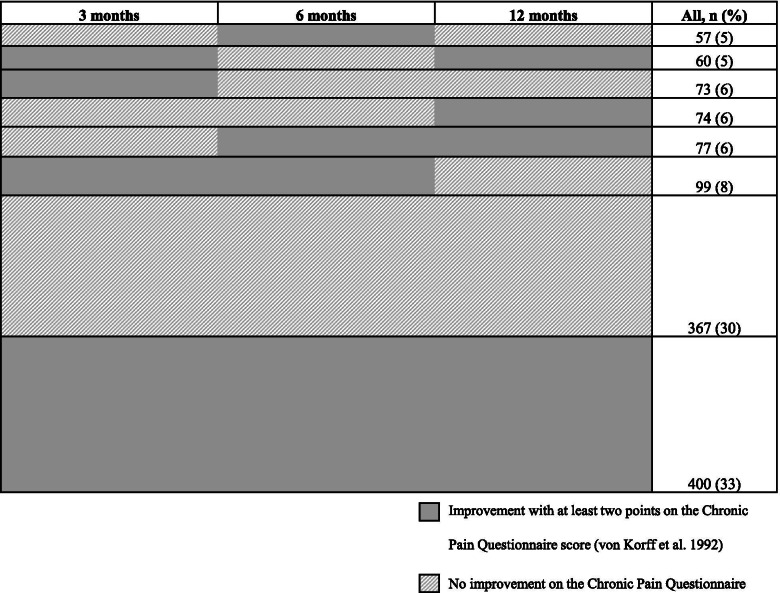
Plot illustrating response patterns of improvement in neck/back pain intensity at follow-up (*n*=1 207)

Figure 2 illustrates outcome patterns for participants responding to all follow-ups (*n* = 1 207). It is presented in consecutive order and the plot shows the proportion of answers, where the majority of participants experienced MCII at all follow-ups.

Table 3 shows crude and adjusted associations between levels of LPA and MCII at 3-, 6- and 12-months. The first section presents the full cohort. Results for exposed participants performing moderate and vigorous LPA were RR 1.07 (95% CI; 0.88-1.30) and 1.19 (95% CI; 0.94-1.51) at 3 months, respectively, compared to the control group. At 6 months, the RR were 1.01 (95% CI; 0.84-1.23) and 1.23 (95% CI; 0.88-1.43) for moderate and vigorous LPA, respectively. At 12 months, the corresponding estimates for moderate and vigorous LPA were RR 1.07 (95% CI; 0.87-1.31) and RR 1.35 (95% CI; 1.06-1.73), respectively. The second section shows the results of the sub-group of those with BP, including fraction of persons with equally disturbing NBP. At the 3-month follow-up, the RR was 1.02 (95% CI; 0.76-1.38) and 1.19 (95% CI; 0.84-1.68) for moderate and vigorous LPA, respectively. At 6-months, corresponding results were 0.98 (95% CI; 0.73-1.31) and 1.12 (95% CI; 0.80-1.57). Finally, at 12-months, those practicing moderate LPA had a 33% greater chance of MCII compared to the control group; RR 1.33 (95% CI; 0.96-1.86) whereas those frequently practicing LPA on a vigorous lever had an 83% greater chance of MCII; RR 1.83 (95% CI; 1.26-2.66). The third section includes those with NP only. Neither moderate nor vigorous LPA were associated with the outcome at any of the follow-ups.

**Table 3:** Associations between leisure-time physical activity and improvement in neck/back pain intensity measured at follow-up

			Crude		Adjusted	
	Cases^**a**^/all	Incidence of improvement (%)	RR	95% CI	RR	95% CI
**Neck and/or back pain (*n*** **= 1 460)**						
**3 months**^**b**^						
Low/irregular^c^	153/308	50	1		1	
Moderate^d^	375/736	51	1.03	0.86-1.25	1.07	0.88-1.30
Vigorous^e^	149/261	57	1.15	0.91-1.44	1.19	0.94-1.51
**6 months**^**f**^						
Low/irregular^c^	155/304	51	1		1	
Moderate^d^	364/728	50	0.99	0.82-1.19	1.01	0.84-1.23
Vigorous^e^	147/260	57	1.11	0.88-1.39	1.23	0.88-1.43
**12 months**^**g**^						
Low/irregular^c^	136/287	47	1		1	
Moderate^d^	336/700	48	1.01	0.83-1.24	1.07	0.87-1.31
Vigorous^e^	152/253	60	1.27	1.01-1.60	1.35	1.06-1.73
**Back pain and back/neck pain combined (*n*** **= 654)**						
**3 months**^**h**^						
Low/irregular^c^	61/123	50	1		1	
Moderate^d^	166/317	52	1.07	0.80-1.44	1.02	0.76-1.38
Vigorous^e^	86/139	62	1.26	0.90-1.75	1.19	0.84-1.68
**6 months**^**i**^						
Low/irregular^c^	64/122	52	1		1	
Moderate^d^	168/312	54	1.03	0.77-1.37	0.98	0.73-1.31
Vigorous^e^	85/135	63	1.20	0.87-1.66	1.12	0.80-1.57
**12 months**^**j**^						
Low/irregular^c^	47/177	27	1		1	
Moderate^d^	159/301	53	1.32	0.95-1.82	1.33	0.96-1.86
Vigorous^e^	91/132	69	1.72	1.21-2.44	1.83	1.26-2.66
**Neck pain (*n*** **= 806)**						
**3 months**^**k**^						
Low/irregular^c^	91/183	50	1		1	
Moderate^d^	209/419	50	1.01	0.91-1.14	1.11	0.86-1.44
Vigorous^e^	62/120	52	1.02	0.91-1.14	1.12	0.80-1.58
**6 months**^**l**^						
Low/irregular^c^	90/180	50	1		1	
Moderate^d^	196/416	47	0.95	0.74-1.22	1.04	0.80-1.34
Vigorous^e^	62/124	50	0.99	0.72-1.37	1.08	0.77-1.52
**12 months**^**m**^						
Low/irregular^c^	89/168	53	1		1	
Moderate^d^	177/399	44	0.84	0.65-1.09	0.93	0.72-1.22
Vigorous^e^	61/120	51	0.95	0.69-1.32	1.06	0.75-1.49

^a^Minimal clinically important improvement in pain intensity defined by improvement on Chronic Pain Questionnaire score by two points or more

^b^Adjusted by age, level of education, pain duration, pain location and treatment arm

^c^No/irregular moderate or vigorous LPA, but any on low exertion level (e.g. calm walks and cycling)

^d^Regular moderate LPA (corresponding to a level that makes it possible to have a conversation) up to three times/week or more, and/or once or twice/week on a vigorous level (high pulse, feeling strained and sweaty)

^e^Regular vigorous LPA three times or more/week with or without LPA on low or moderate level

^f^Adjusted by age, level of education, pain duration, mean disability at baseline, pain location and treatment arm

^g^Adjusted by age, level of education, pain duration, mean disability at baseline, body mass index, pain location and treatment arm

^h^Adjusted for age, pain duration and treatment arm

^i^Adjusted for pain duration and treatment arm

^j^Adjusted for age, pain duration and treatment arm

^k^Adjusted for age, sex, education, daily smoking, body mass index, pain duration, previous episodes of pain, baseline pain intensity, baseline disability and treatment arm

^l^Adjusted for age, sex, education, body mass index, pain duration, previous episodes of pain, baseline pain intensity, baseline disability and treatment arm

^m^Adjusted for age, sex, education, body mass index, pain duration, previous episodes of pain, baseline pain intensity and treatment arm

### Sensitivity analysis

The analysis of participants responding to all follow-ups (*n* = 767) and had either MCII at all follow-ups (*n* = 400) or at no follow-up (*n* = 367) yielded similar results as the main 12-month analyses; RR 1.04 (95% CI; 0.80-1.35) for moderate LPA and RR 1.28 (95% CI; 0.94-1.73) for vigorous LPA, respectively. This was done for the full cohort only.

## Discussion

This study suggests that persons with NBP who have received manual therapy or general evidence-based care have a greater chance of MCII if they prior to treatment frequently practice LPA on a vigorous level. This is in comparison to persons with NBP who never or irregularly practice LPA on a moderate or vigorous level. The association is seen at the 12-month follow-up after treatment, but not earlier during follow-up. Stratified analyses showed an effect only among those with mainly BP. There was also a tendency that those with mainly BP who practice LPA on moderate level had a better chance of MCII than those who never or irregularly practiced LPA, although not statistically significant.

A recent systematic review assessing the current evidence about the influence of physical activity on management of neck and back pain, did not find any studies concerning prognosis [27]. Our results are to some extent in line with results from a study by Pinto et al, suggesting that involvement in moderate-to-vigorous LPA resulted in an improvement in low BP after 12 months, compared to those with less/no LPA [14]. The results are also in line with a study based on secondary analysis by Hurwitz et al., where self-reported weekly LPA was converted into four categories of metabolic equivalent of task (METs). They found a tendency to dose-response relation where those with highest METs had decreased low BP [28]. Nonetheless, a thorough systematic review conclude that the evidence is low, pointing towards no associations [15]. Our results of nil-findings for NP are also in line with the majority of previous literature [7].

There are several possible reasons for the current conflicting results. Misclassification of LPA is possible, especially as it often is based on self-reported data with few details of frequency and intensity. Also, most studies have dichotomized the exposure to either being involved in LPA or not. Furthermore, there is often no consideration of reversed causation (i.e. pain prevents persons from being physically active). These circumstances would most likely dilute any true effect in the association between LPA and recovery from NP and BP.

The mechanisms behind our findings can only be speculated. It has been suggested that physiological effects of LPA are beneficial for recovery from pain conditions [8, 9]. However, we did not find an increased chance of improvement among those who reported regular LPA on a moderate intensity level, except for BP after 12 months, and the effect was modest. This might be a result of exposure misclassification, either due to the nature of self-report, or the way we categorized moderate LPA. Furthermore, our unexposed group also included persons who frequently took slow walks and participated in other non-vigorous LPA, which, if these activities also are favorable for pain improvement from NBP, may have diluted our findings in both exposure groups. Another explanation for our results might be that practicing frequent and vigorous LPA may be a proxy for having a structured personality trait with good compliance to advices, generally given by the therapists in the trials, and that the effect actually may be a result of a successful intervention. On the other hand, this would likely be true for NP patients as well. The musculoskeletal system in the back is most often involved in LPA no matter what type of LPA you practice. For the musculoskeletal system of the neck, this might not be the case. Important to note is also that the causal relationship between LPA and improvement in NP and BP (the mechanism) is yet to be confirmed.

### Strengths

The study has several strengths. It is a relatively large study with less than 20% loss to follow-up over 12 months. The outcome measurement is reliable and frequently used and considered to mirror clinically meaningful changes over time [22, 23]. The way the exposure was categorized captures both frequency and level of exertion of LPA, which may be one reason to why we found an association, when most previous studies on this topic, has not [7, 15, 29].

One common bias in studies investigating the importance of LPA for recovery from musculoskeletal conditions is the risk of reversed causation. That is, if persons avoid LPA due to worsening in pain when being physically active, or that pain prevents them from being physically active. If so, there would be an inverse relation or at least a dilution of a true effect between LPA and recovery. A strength in our study is the high number of potential confounders considered, as baseline pain characteristics. Level of disability and duration of pain was considered confounders and were included in the adjusted models, as were baseline pain intensity in some of the models. Unfortunately, we did not have statistical power to stratify the analysis by pain duration or level of disability, to deepen the knowledge about these associations.

### Limitations

As discussed above, the exposure may be prone to non-differential misclassification bias. If so, this would most likely have resulted in a dilution of the findings, however not different between persons with NP and BP, thus it cannot explain differences found between the pain locations. Moreover, despite a relatively large study population, we did not have statistical power to stratify by pain duration or disability, speaking towards variability in the data. It is possible that these factors also might act as effect modifiers, i.e. LPA has different effect on pain improvement across strata. Unmeasured confounders such as psychological distress, or other psychological factors often concurring with NBP, may lead to over- or underestimation. Furthermore, another unmeasured potential confounder is occupational physical activity [30].One previous study with sickness absence as the outcome, found that moderate or high occupational physical activity was associated with sickness absence for more than three weeks, whereas LPA was associated with a decreased risk of sickness absence (diagnoses not specified) [31]. If this would be the case also for prognosis of neck and back pain as in our study, we are likely to have under- or overestimated our results, depending on the co-occurrence of leisure time physical activity and occupational physical activity in our study population. However, in a previous study we found that occupational physical activity was not associated with neither the prognosis, nor the risk of onset of neck pain [16]. Nevertheless, we cannot rule out the importance of this factor for the associations in the present study.

### Generalizability

Patients agreeing to participate in an RCT may differ from patients refusing participation, which possibly could affect the generalizability of the study results. However, in order to create a selection bias, the preference must relate to both exposure and outcome [26]. Common factors involved in non-participation are male gender and younger age, as well as smoking and low LPA [32]. If those who refused participation more often had low LPA, and also had a better chance to improve in pain intensity than those with low LPA who participated, we may have overestimated the results due to selection bias. In summary, we believe that the results are generalizable to other populations aged 18-65 who have received manual therapy or general care for NP and BP. Nevertheless, future studies need to be of sufficient size to allow stratification by sub-groups such as pain duration and pain disability, since such factors may modify the effect of LPA on recovery from NBP.

## Conclusions

This study suggests that persons with NBP in a working population, who have received manual therapy or general evidence-based care, have a greater chance of MCII in pain intensity after 12 months if they prior to treatment frequently practice vigorous LPA. When analyzed separately on pain location, the effect was only present in persons with BP.

## Data Availability

The datasets generated and/or analyzed during the current study are not publicly available due to ethical restrictions and laws of disclosing personal data but are available from the corresponding author on reasonable request. Data will be available upon request after ethical permission.

## Abbreviations

BP: Back pain
CPQ: Chronic Pain Questionnaire
LPA: Leisure-time physical activity
MCII: Minimal clinically important improvement
MINT: Stockholm Manual Intervention Trial
NBP: Neck and back pain
NP: Neck pain
